# Distinct Changes in Global Brain Synchronization in Early-Onset vs. Late-Onset Parkinson Disease

**DOI:** 10.3389/fnagi.2020.604995

**Published:** 2020-12-14

**Authors:** Tianyu Wang, Haiyan Liao, Yuheng Zi, Min Wang, Zhenni Mao, Yijuan Xiang, Lin Zhang, Junli Li, Qin Shen, Sainan Cai, Changlian Tan

**Affiliations:** ^1^Department of Radiology, The Second Xiangya Hospital, Central South University, Changsha, China; ^2^Department of Radiology, Hunan Province Maternal and Child Health Care Hospital, Changsha, China

**Keywords:** early-onset Parkinson disease, late-onset Parkinson disease, global brain synchronization, degree centrality, resting-state functional MRI (R-fMRI)

## Abstract

Early- and late-onset Parkinson’s disease (EOPD and LOPD, respectively) have different risk factors, clinical features, and disease course; however, the functional outcome of these differences have not been well characterized. This study investigated differences in global brain synchronization changes and their clinical significance in EOPD and LOPD patients. Patients with idiopathic PD including 25 EOPD and 24 LOPD patients, and age- and sex-matched healthy control (HC) subjects including 27 younger and 26 older controls (YCs and OCs, respectively) were enrolled. Voxel-based degree centrality (DC) was calculated as a measure of global synchronization and compared between PD patients and HC groups matched in terms of disease onset and severity. DC was decreased in bilateral Rolandic operculum and left insula and increased in the left superior frontal gyrus (SFG) and precuneus of EOPD patients compared to YCs. DC was decreased in the right putamen, mid-cingulate cortex, bilateral Rolandic operculum, and left insula and increased in the right cerebellum-crus1 of LOPD patients compared to OCs. Correlation analyses showed that DC in the right cerebellum-crus1 was inversely associated with the Hamilton Depression Scale (HDS) score in LOPD patients. Thus, EOPD and LOPD patients show distinct alterations in global synchronization relative to HCs. Furthermore, our results suggest that the left SFG and right cerebellum-crus1 play important roles in the compensation for corticostriatal–thalamocortical loop injury in EOPD and LOPD patients, whereas the cerebellum is a key hub in the neural mechanisms underlying LOPD with depression. These findings provide new insight into the clinical heterogeneity of the two PD subtypes.

## Introduction

Idiopathic Parkinson’s disease (PD) can be subdivided into early- and late-onset forms (EOPD and LOPD, respectively) according to the age of onset (before or after 50 years, respectively; Schrag and Schott, 2006; Marras and Lang, 2013). The two subtypes of PD differ in terms of risk factors, clinical features, and disease course. For example, genetic factors have a greater influence on the occurrence of EOPD compared to LOPD, with first-degree relatives of EOPD patients more frequently exhibiting nonmotor symptoms and being at higher risk of developing PD (Stern et al., 1991; Liu et al., 2018). On the other hand, LOPD tends to progress more rapidly and patients are more likely to have gait disorder and dystonia, although EOPD is associated with a higher incidence of motor complications (Inzelberg et al., 2004; Wickremaratchi et al., 2009). LOPD patients typically present with more nonmotor symptoms that are also of greater severity than EOPD patients (Guo et al., 2013), but depression (Knipe et al., 2011; Mehanna et al., 2014) and loss of vision (Feitosa-Santana et al., 2020) are more common in EOPD. Histopathologic and molecular analyses have also revealed a greater loss of dopaminergic neurons in the substantia nigra and damage to the dopaminergic system in EOPD patients (Mayer et al., 1986; Shih et al., 2007). These findings suggest that distinct neuropathophysiologic mechanisms underlie EOPD and LOPD.

Neuroimaging is useful for investigating differences between EOPD and LOPD in terms of brain structure, iron deposition, and functional impairment. For instance, neuroimaging combined with quantitative susceptibility mapping revealed similar changes in iron content in the substantia nigra pars reticulata and pars compacta in EOPD and LOPD, but greater iron deposition in the putamen in LOPD (Xuan et al., 2017a). A voxel-based morphometry study reported different degrees of gray matter density changes in multiple brain regions in the two PD subtypes, including the frontal cortex and posterior lobes of the cerebellum (Xuan et al., 2017b). In the last 5 years, blood oxygenation level-dependent resting-state functional magnetic resonance imaging (rs-fMRI) has become a commonly used research instrument for exploring changes in spontaneous brain activity in PD (Fox and Raichle, 2007; Van Eimeren et al., 2009). Functional neuroimaging studies of EOPD and LOPD have mostly focused on spontaneous brain activity as measured by regional homogeneity (ReHo; Sheng et al., 2016; Yue et al., 2020), the amplitude of low-frequency fluctuation (ALFF; Yue et al., 2020), and functional connectivity (FC; Hou et al., 2016). However, there are many inconsistencies in the findings, and it remains unclear which neuroimaging markers are more reliable for evaluating the pathophysiologic and clinical differences between EOPD and LOPD. It has been suggested that the two PD subtypes have distinct pathophysiologic mechanisms that are attributable to dysfunction in specific brain network regions (loops). However, the abovementioned rs-fMRI parameters provide limited information: ReHo and ALFF are both functional segregation methods that reflect local functional alterations but not the strength of functional connections between different brain regions, while FC requires an *a priori* hypothesis, which can lead to selection bias due to subjective factors (Lv et al., 2018).

Degree centrality (DC) is a reliable rs-fMRI parameter (Zuo et al., 2013) that does not require any modal assumptions and can reveal whole-brain FC strength at the voxel level (i.e., FC density or global synchronization; Buckner et al., 2009; Tomasi and Volkow, 2012; Zuo et al., 2012). Previous studies have shown that changes in cognition and motor function in PD are reflected by alterations in global synchronization (Li et al., 2019), whereas extensive changes in DC have also been observed in PD with depression (Wang et al., 2018; Zhang et al., 2018). In the present study, we used DC as a neuroimaging marker to investigate differences in global brain synchronization between EOPD and LOPD. Our findings provide insight into the neural mechanisms underlying the clinical heterogeneity of the two PD subtypes.

## Materials and Methods

### Subjects

The study protocol was approved by the Medical Ethics Committee of the Second Xiangya Hospital, Central South University. Before enrollment, written, informed consent was obtained from all participants or their guardians to ensure voluntary participation.

Patients with idiopathic PD were recruited from the Department of Neurology, the Second Xiangya Hospital of Central South University from January 2016 to February 2020. PD was diagnosed by two experienced neurologists according to the UK PD Society Brain Bank criteria (Hughes et al., 1992). The inclusion criteria were as follows: (1) in an “off ” state and Hoehn–Yahr stage ≤3.0; (2) cognitive ability not lower than the corresponding education level according to Mini-Mental State Examination (MMSE) score (Li et al., 2016; i.e., no apparent cognitive impairment); (3) right-handedness; and (4) no history of other neurologic or psychiatric illnesses. According to the age of onset [before or after the age of 50 years (Schrag and Schott, 2006)], PD patients were divided into two groups: EOPD (*n* = 25; median age: 47.64 ± 2.67 years; 11 male) and LOPD (*n* = 24; median age: 62.38 ± 5.14 years, 18 male). Two age- and sex-matched groups of healthy control subjects (HCs) were enrolled from the local community: younger controls (YCs) matched to EOPD (*n* = 27; median age: 48.84 ± 3.05 years; eight male) and older controls (OCs) matched to LOPD (*n* = 26; median age: 62.54 ± 6.92 years; 15 male).

Before the MRI scan, all participants were assessed with the MMSE, Unified Parkinson Disease Rating Scale (UPDRS), and Hamilton Depression Scale (HDS). And the Daily Levodopa Equivalents Dose (DLED) of the medicated patients were also calculated (Tomlinson et al., 2010). The demographic and clinical data of all participants are shown in Table 1.

**Table 1:** Demographic and clinical characteristics of Parkinson disease patients and healthy control subjects.

	EOPD	YC	LOPD	OC	*p*-value		
EOPD vs. YC	LOPD vs. OC	EOPD vs. LOPD					
Sample size (male)	25 (11)	27 (8)	24 (18)	26 (15)	0.091	0.242	0.232
Age, years	47.64 ± 2.67	48.85 ± 3.05	62.38 ± 5.14	62.54 ± 6.92	0.134	0.925	<0.001
Age of onset, years	45.30 ± 2.51	NA	59.81 ± 4.99	NA			<0.001
Disease duration, months	28.08 ± 20.10	NA	35.75 ± 25.85	NA			0.254
Education, years	7.52 ± 2.62	8.51 ± 2.73	6.12 ± 3.18	7.46 ± 3.64	0.185	0.172	0.101
DLED, mg	173.33 ± 235.18	NA	208.82 ± 190.59	NA			0.646
H-Y stage	1.64 ± 0.62	NA	1.92 ± 0.72	NA			0.277
UDPRS	24.68 ± 13.72	NA	34.25 ± 19.92	NA			0.058
UDPRS III	15.00 ± 7.58	NA	21.25 ± 13.46	NA			0.055
MMSE	27.56 ± 2.55	28.04 ± 2.29	26.12 ± 2.64	24.85 ± 4.60	0.483	0.231	0.059
HDS	6.44 ± 6.26	1.30 ± 1.86	9.17 ± 6.82	2.61 ± 2.80	<0.001	<0.001	0.152

*All data are presented as median ± SD. EOPD, early-onset Parkinson disease; LOPD, late-onset Parkinson disease; YC, younger control; OC, older control; DLED, Daily Levodopa Equivalent Dose; H-Y, Hoehn, and Yahr; UDPRS, Unified Parkinson’s Disease Rating Scale; MMSE, Mini-Mental State Examination; HDS, Hamilton Depression Scale*.

### MR Data Acquisition and Preprocessing

All MR data were obtained on a 3.0 T MRI scanner (MAGNETOM Skyra; Siemens Healthineers, Erlangen, Germany). High-resolution T1-weighted transverse images were acquired as a volumetric three-dimensional magnetization-prepared rapid gradient-echo sequence [176 axial slices; slice thickness = 1.0 mm (no slice gap), repetition time (TR) = 1,900 ms, echo time (TE) = 2.01 ms, flip angle (FA) = 9°, field of view (FOV) = 256 × 256 mm^2^]. Functional images were obtained by echo-planar imaging [EPI; 39 axial slices; slice thickness = 3.5 mm (no slice gap), TR = 2,500 ms, TE = 25 ms, FA = 90°, FOV = 240 × 240 mm^2^; matrix size = 64 × 64, voxel size = 3.8 × 3.8 × 3.5 mm^3^; 200 volumes]. Participants were instructed to remain relaxed with their eyes closed but without falling asleep during the scan; a foam cushion was used to reduce head movement and participants wore earplugs to minimize noise.

Data preprocessing was carried out using the Resting-State fMRI Data Analysis Toolkit (RESTplus) v1.21 (Jia et al., 2019)^1^ as previously described (Liao et al., 2020; Wang et al., 2020). Briefly, the first 10 time points from each subject’s series were discarded; this was followed by realignment and correction for head motion (exclusion criteria: head movement >0.5 mm or angular rotation >0.5° in any direction), co-registration between T1 and EPI images, spatial normalization to a standard Montreal Neurological Institute template, and voxel resampling to a voxel size of 3 × 3 × 3 mm^3^. Linear trends and nuisance signals (including head motion and white matter and cerebrospinal fluid signals) were removed, followed by denoising and bandpass (0.01–0.08 Hz) filtering.

### DC Calculation

DC maps were generated for each subject using RESTplus software as previously described (Buckner et al., 2009; Zuo et al., 2012). Briefly, we computed individual Pearson’s correlation coefficients in *a prior* probability brain gray matter mask in Statistical Parametric Mapping eight^2^ between the time course of a given voxel and all other whole-brain voxels within the template. We then generated a whole-brain FC matrix for each subject. As different thresholds can impact the results, the correlation threshold was set to 0.25 in accordance with previous studies (Buckner et al., 2009; Zuo et al., 2012). The calculated individual correlation matrix was standardized by Fisher’s *r*-to-*z* transformation for individual weighted DC mapping, and spatial smoothing was performed by applying a 6-mm full width at half-maximum Gaussian smoothing kernel.

### Statistical Analysis

Statistical analysis was performed using SPSS v22.0 software (SPSS Inc., Chicago, IL, USA). The two-sample *t*-test was used to evaluate differences between DC and other demographic parameters in pairwise comparisons (EOPD vs. YC and LOPD vs. OC). The statistical significance threshold was set at *p* < 0.001, combined with voxel size >15 corresponding to a corrected *p* < 0.05, which was determined using the AlphaSim tool in RESTplus software. Correlations between clinical data and DC within clusters that showed significant differences in previous analyses were assessed with the Spearman correlation coefficient. Significance threshold was set at *p* < 0.001 voxel-level uncorrected and *p* < 0.05 cluster level FDR corrected.

## Results

### Demographics and Clinical Characteristics of the Study Population

Demographic and clinical characteristics of the study population are shown in Table 1. Apart from median age and age of onset, EOPD and LOPD groups had similar profiles including disease duration and severity and clinical assessment scale scores. The HDS scores of EOPD and LOPD patients were higher than those of the respective age-matched control groups (*p* ≤ 0.001), whereas no significant differences were observed in terms of age, sex, education level, DLED, and MMSE score.

### Differences in Global Synchronization

**Figure 1:**
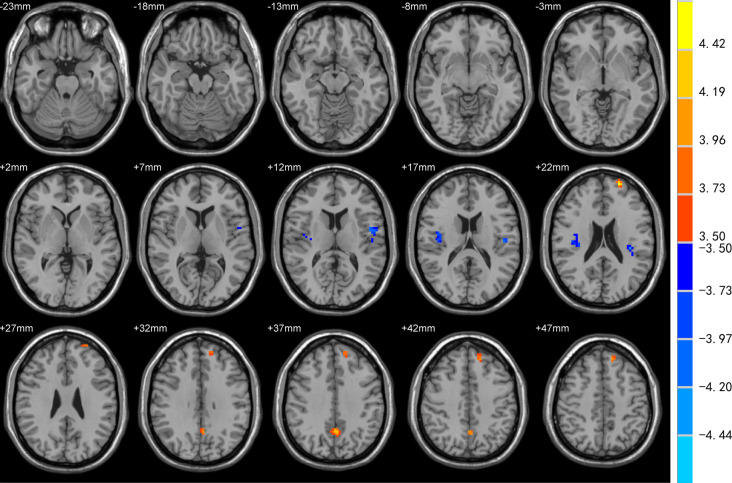
Comparison of degree centrality (DC) between EOPD and YC groups. DC was increased in the left superior frontal gyrus (SFG), precuneus, and decreased in bilateral Rolandic operculum and left insula in the EOPD group (all *p* < 0.05).

**Figure 2:**
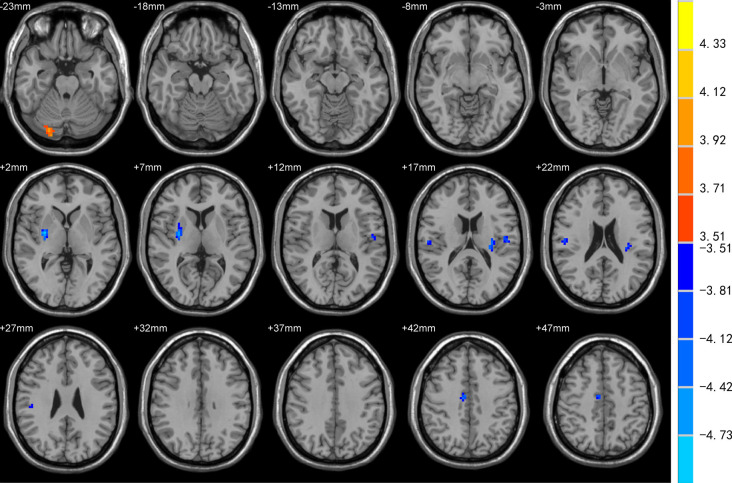
Comparison of DC between LOPD and OC groups. DC was increased in the right cerebellum-crus1 and decreased in the right putamen, mid-cingulate cortex, bilateral Rolandic operculum, and left insula in the LOPD group (all *p* < 0.05).

DC was increased in the left superior frontal gyrus (SFG), precuneus, and decreased in bilateral Rolandic operculum and left insula of EOPD patients compared to YC subjects (Figure 1). DC was increased in the right cerebellum-crus1 and decreased in the right putamen, mid-cingulate cortex, bilateral Rolandic operculum, and left insula of the LOPD group compared to the OC group (Figure 2). Table 2 lists the local maxima of DC values determined with the two-sample *t*-test.

**Table 2:** Degree centrality analysis of altered brain regions in functional magnetic resonance imaging in early- and late-onset Parkinson disease patients and sex-and age-matched healthy control subjects*.

Brain region	Peak MNI coordinates			Voxel size	*T*-value
	*X*	*Y*	*Z*		
EOPD < YC					
Rolandic_Oper_L	−51	0	9	33	−4.334
Insula_L	−36	−27	21	15	−4.205
Rolandic_Oper_R	42	−9	15	46	−4.677
EOPD > YC					
Frontal_Sup_L	−21	63	24	21	4.646
Frontal_Sup_L	−12	42	45	32	4.228
Precuneus_L	−3	−57	36	31	4.355
LOPD > OC					
Cerebelum_Crus1_R	24	−84	−24	21	3.995
LOPD < OC					
Putamen_R	30	−6	3	34	−4.906
Insula_L	−30	−21	18	19	−4.327
Rolandic_Oper_R/Postcentral Gyrus	45	−21	24	25	−4.293
Rolandic_Oper_L/Postcentral Gyrus	−51	−12	15	17	−4.442
Cingulum_Mid_R	6	−9	45	23	−5.025

MNI, Montreal Neurological Institute. *The *p*-value was < 0.001 and voxel size was larger >15 (*p* < 0.05, AlphaSim-corrected).

### Correlation Analysis

**Figure 3:**
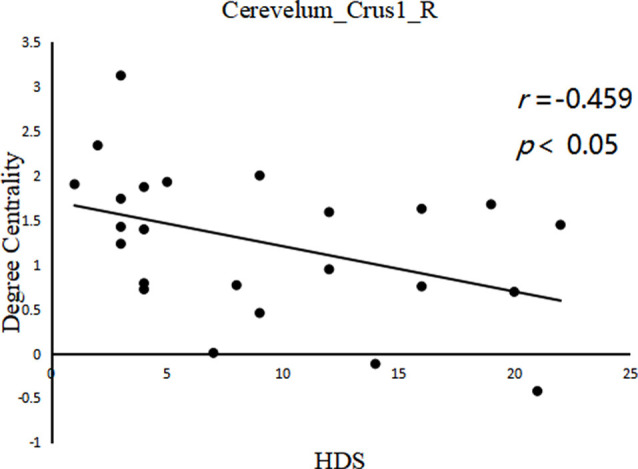
Between cerebelum_crus1_R (*x, y, z* = 24, −84, −24) DC value and hamilton depression scale (HDS) score in the LOPD group (*r* = −0.459, *p* = 0.024, FDR cluster level corrected, starting from voxel level *p* < 0.001 uncorrected).

A significant negative correlation was observed between DC of the right cerebellum_crus1 (*x, y, z* = 24, –84, –24) and HDS score in LOPD patients (*r* = −0.459, *p* = 0.024, FDR cluster level corrected, starting from voxel-level *p* < 0.001 uncorrected; Figure 3). There were no other significant correlations between clinical data and DC in other clusters in PD patients.

## Discussion

The present study used DC as an indicator of differences in spontaneous brain activity (specifically, in global synchronization) between EOPD and LOPD patients. We investigated the correlation between clinical data and global synchronization in brain regions with significant differences between PD patients and healthy control (HC) subjects (EOPD vs. YC and LOPD vs. OC) based on the classic model of corticostriatal-thalamocortical (CSTC) loop dysfunction in PD (Helmich et al., 2010; Hacker et al., 2012). We also compared changes in the brain network [default mode network (DMN) and cingulo-opercular network (CON)] between the two subtypes of PD.

The LOPD group showed decreased global synchronization in the right putamen, which is a key node of the CSTC loop that is damaged in PD (Ryoo et al., 2010) and is more closely related to disease frequency and severity than the caudate (Sasannezhad et al., 2017). The putamen was shown to be hypoactive and have lower blood flow in PD patients than in normal subjects (Playford et al., 1992; Rowe et al., 2002), and structural MRI and fMRI studies have revealed decreased gray matter volume (Xuan et al., 2017b) and reduced regional synchronization (Sheng et al., 2016). Thus, a decrease in global synchronization may be a manifestation of putamen injury in PD.

We also observed increased global synchronization in the right cerebellum_crus1. The cerebellum is involved in sensorimotor processing (Kansal et al., 2017) and cognitive-emotional processing (Adamaszek et al., 2017); functional and structural changes in this region have been linked to gait disturbance, muscular rigidity, and some nonmotor symptoms of PD (Wu and Hallett, 2013). The cerebellum influences motor and cognitive functions *via* cerebello-thalamocortical circuits (Middleton and Strick, 2001). Cognitive function as measured by Frontal Assessment Battery score was positively correlated with DC of the cerebellum, which was in turn negatively correlated with total UPDRS scores, implicating both the cognitive and motor functions of the cerebellum in PD pathophysiology (Li et al., 2019). The cerebellum projects extensively to the putamen *via* the thalamus (Hoshi et al., 2005); previous fMRI studies showed that higher putamen-cerebellar FC was correlated with better motor performance (Simioni et al., 2016) and that there was a greater compensatory increase in FC in the cerebellum-putamen circuit in LOPD patients than in EOPD patients (Hou et al., 2016). Our results suggest that increased global synchronization in the cerebellum may compensate for the striatal dysfunction (mainly in the putamen) in LOPD. This could explain why the incidence of motor complications is lower in LOPD than in EOPD (Alves et al., 2005; Schrag and Schott, 2006). Moreover, we observed a negative correlation between DC of the right cerebellum_crus1 and HDS score in LOPD. This result is inconsistent with the previous finding that DC was decreased in the cerebellum of PD patients with depression compared to those without depression, although the patients were not subdivided by age of onset (Wang et al., 2018). Our results imply that the cerebellum is a key node in the neural network underlying LOPD with depression.

DC was increased in the left SFG of EOPD patients compared to YCs. The frontal gyrus is not only engaged in the control of motion (Tanei et al., 2009) but is also related to attention to the movement (Jueptner et al., 1997). It was proposed that overactivation of the SFG is a compensatory mechanism in EOPD (Long et al., 2012), which may also be characterized by altered regional volume and cerebral blood flow in this area (Yang et al., 2013). Overactivation of the prefrontal cortex (PFC) was shown to decrease over time in EOPD patients, which may be associated with disease progression (Baglio et al., 2011). PD is thought to involve the dysfunction of corticostriatal pathways, which include parallel and independent circuits such as limbic, sensorimotor, and associative circuits (Alexander et al., 1986). The latter two circuits control habits and goal-directed behaviors (Graybiel, 2008; Balleine and O’Doherty, 2010), and dysfunction of the sensorimotor circuit is a major cause of movement disorder in PD (Grafton, 2004). Compared to LOPD patients, EOPD patients exhibit increased FC in the caudate nucleus-sensorimotor loop to offset motor dysfunction (Hou et al., 2016). Frontostriatal-thalamic pathways mediate motor response inhibition (Jahanshahi et al., 2015). Associative circuits in the corticostriatal pathway project from associative cortical areas (e.g., PFC-SFG) to the putamen and caudate nucleus (Baglio et al., 2011). Thus, the increased global synchronization in the left SFG may reflect hyperactivation of the associative circuit to counter the inhibition of basal ganglia in the CSTC loop in EOPD patients.

The frontal gyrus is implicated in emotion and executive and cognitive functions (Jueptner et al., 1997; Miller and Cohen, 2001). The medial PFC, together with the precuneus and cingulate cortex are critical brain regions in the DMN (Raichle et al., 2001; Laird et al., 2009). In our study, DC was increased in the left SFG and precuneus of EOPD patients and decreased in the right mid-cingulate cortex of LOPD patients, suggesting opposite global synchronization changes in important DMN regions in the two PD subtypes. Task-negative cerebral activity is a function of the DMN; that is, the network is activated in the resting state and deactivated during task performance (Raichle et al., 2001). Higher FC of the DMN in PD may lead to a decline in the capacity for self-referential processing, a greater propensity to remain in the baseline mode, and reduced control of interactions between different brain regions (Li et al., 2019). A decrease in DMN FC has been linked to cognitive impairment (Lucas-Jiménez et al., 2016), while increased intrinsic DMN activity was shown to be a compensatory mechanism in mild cognitive impairment (Liang et al., 2020). Several fMRI studies have demonstrated increased FC between brain regions of the DMN in depressed PD patients (Hu et al., 2015; Lou et al., 2016). We speculate that the increased global synchronization of critical DMN brain regions in EOPD is responsible for the higher incidence of depression but better cognitive function in EOPD patients compared to LOPD patients matched in terms of disease duration and severity (Schrag and Schott, 2006;Spica et al., 2013).

Compared to YCs, EOPD patients showed decreased DC in bilateral Rolandic operculum and left insula, whereas LOPD patients also showed decreased DC in the bilateral Rolandic operculum and left insula relative to OCs. These brain regions are all components of the CON, which is involved in task initiation, maintenance, and monitoring (Dosenbach et al., 2008). In a previous study of Parkinson-related CON alterations, graph theory-based network analysis revealed that PD patients showed decreased node strength and betweenness centrality in CON-related nodes (Chen et al., 2015). Functional separation and integration deficiency between the CON and DMN have also been reported, which could lead to difficulties in task initiation and maintenance (Tinaz et al., 2016). However, additional studies are needed to clarify the differences between EOPD and LOPD concerning changes in the CON.

This study had some limitations. First, the sample size was relatively small; follow-up studies with a larger cohort involving multiple research institutions would provide more reliable conclusions. Second, though the correlation between clinical data and DC within clusters that showed significant differences was reached the medium level, the significance cannot survive the strict Bonferroni correction. This might be related to our relatively small sample while the narrow range of clinical data also limited the correlation analysis. Future analysis conducted in larger samples and more sufficient clinical data are warranted. Third, the patients in our study were not drug-naïve; although they were required to cease taking anti-PD medications for 12 h before MRI scanning and neuropsychologic testing, the residual effects of medications may have biased the results. Last but not least, though the AlphaSim correction of *p* < 0.05 with a combined individual voxel threshold at *p* < 0.001 and the required cluster size adopted in the present study was acceptable based on the previous studies (Fan et al., 2017; Fu et al., 2019), it was not that strict to some extent. Thus, we might suggest that it should be cautious to generalize our results and further replication studies are needed.

In summary, the results of this study demonstrate that EOPD and LOPD exhibit distinct alterations in global brain synchronization. The left SFG and right cerebellum_crus1 play central roles in the compensation for CSTC circuit injury in EOPD and LOPD patients, respectively, whereas the cerebellum may serve as a key hub in the neural network underlying LOPD with depression. The different patterns of global synchronization changes in critical regions of the DMN and CON provide insight into the neural basis for the clinical heterogeneity of PD subtypes.

## Data Availability Statement

The raw data supporting the conclusions of this article will be made available by the authors, without undue reservation.

## Ethics Statement

The studies involving human participants were reviewed and approved by Medical Ethics Committee of the Second Xiangya Hospital, Central South University. The patients/participants provided their written informed consent to participate in this study.

## Author Contributions

TW, YZ, ZM, LZ, JL, and YX: data collection. TW, YZ, MW, QS, SC, and ZM: data analysis. TW and HL: manuscript writing. CT: project development and manuscript revision. All authors contributed to the article and approved the submitted version.

## Conflict of Interest

The authors declare that the research was conducted in the absence of any commercial or financial relationships that could be construed as a potential conflict of interest.

## Footnotes

^1^http://www.restfmri.net

^2^http://www.fil.ion.ucl.ac.uk/spm/
